# Colorimetric Aptasensor Using Unmodified Gold Nanoparticles for Homogeneous Multiplex Detection

**DOI:** 10.1371/journal.pone.0109263

**Published:** 2014-10-03

**Authors:** Shucao Niu, Zhenzhen Lv, Jinchuan Liu, Wenhui Bai, Shuming Yang, Ailiang Chen

**Affiliations:** 1 Institute of Quality Standards & Testing Technology for Agro-Products, Key Laboratory of Agro-product Quality and Safety, Chinese Academy of Agricultural Sciences, Beijing, China; 2 Key Laboratory of Agri-Food Quality and Safety, Ministry of Agriculture, Beijing, China; University of Houston, United States of America

## Abstract

Colorimetric aptasensors using unmodified gold nanoparticles (AuNPs) have attracted much attention because of their low cost, simplicity, and practicality, and they have been developed for various targets in the past several years. However, previous research has focused on developing single-target assays. Here, we report the development of a homogeneous multiplex aptasensor by using more than one class of aptamers to stabilize AuNPs. Using sulfadimethoxine (SDM), kanamycin (KAN) and adenosine (ADE) as example targets, a KAN aptamer (750 nM), an SDM aptamer (250 nM) and an ADE aptamer (500 nM) were mixed at a 1∶1∶1 volume ratio and adsorbed directly onto the surface of unmodified AuNPs by electrostatic interaction. Upon the addition of any of the three targets, the conformation of the corresponding aptamer changed from a random coil structure to a rigid folded structure, which could not adsorb and stabilize AuNPs. The AuNPs aggregated in a specific reaction buffer (20 mM Tris-HCl containing 20 mM NaCl and 5 mM KCl), which led to a color change from red to purple/blue. These results demonstrate that the multiplex colorimetric aptasensor detected three targets simultaneously while maintaining the same sensitivity as a single-target aptasensor for each individual target. The multiplex aptasensor could be extended to other aptamers for various molecular detection events. Due to its simple design, easy operation, fast response, cost effectiveness and lack of need for sophisticated instrumentation, the proposed strategy provides a powerful tool to examine large numbers of samples to screen for a small number of potentially positive samples containing more than one analyte, which can be further validated using sophisticated instruments.

## Introduction

Gold nanoparticles (AuNPs) have received tremendous attention in colorimetric biosensors for possessing size- and distance-dependent optical properties. The color of the colloidal gold solution changes from red to purple/blue during AuNP aggregation or from purple/blue to red during redispersion of AuNP aggregates due to varying interparticle plasmon coupling and the resulting surface plasmon band shift. Furthermore, because the extinction coefficient of AuNPs is over 1000 times higher than that of organic dyes [1], the AuNP-based colorimetric recognition shows fairly high sensitivity [2]–[4]. Therefore, the assembly/disassembly of nanoparticles can be used as a novel indicator for colorimetric assays. In 2004, Rothberg et al found that single- and double-stranded oligonucleotides have different adsorption properties on unmodified gold nanoparticles in colloidal solution, and ssDNA can electrostatically adsorb onto the surface of AuNPs and stabilize the gold nanoparticles against aggregation at certain salt concentrations [5]. Based on this principle, a hybridization assay was designed to detect DNA sequences based on the color changes associated with gold aggregation.

Aptamers are short ssDNAs or RNAs selected with a combinatorial technique called Systematic Evolution of Ligands by EXponential enrichment (SELEX). As an emerging class of recognition elements, aptamers can bind a broad range of targets, from metal ions and organic molecules to proteins and cells, with high specificity and affinity. In comparison with their antibody equivalents, aptamers possess several advantages for the construction of biosensors, such as cost-effective synthesis, temperature stability, consistency between different lots, and flexibility for signal transduction and detection [6]. Moreover, aptamers possess a special ability to undergo target-induced conformational change from free uncoiled ssDNAs to folded structures with higher rigidity. This property is the basis of many sensing approaches, including fluorescent methods, colorimetric methods [7], [8], and electrochemical methods [9], [10].

By integrating the strengths of both AuNPs and aptamers, Fan et al developed the first AuNP-based colorimetric aptasensor for potassium ion detection [11]. In this method, the potassium aptamer was adsorbed onto the surface of unmodified AuNPs in the colloidal gold solution, which remained red in color at a high salt concentration. Upon the addition of potassium, the conformation of aptamers changes to a folded state and is desorbed from the surface of AuNPs. Subsequently, AuNPs aggregate, and the color of the colloidal gold solution changes to purple/blue. Recently, the AuNP-based colorimetric aptasensor has been one of the most commonly used methods for the design of aptamer-based assays, primarily due to the ease of detection, high sensitivity, and potential for high-throughput analysis [12]–[18].

However, all of the above-mentioned AuNP-based colorimetric aptasensors use single target detection; therefore, the development of a multiplex detection utilizing the AuNP-based colorimetric aptasensor is highly desirable. Because the underlying adsorption mechanism of an aptamer onto the surface of unmodified AuNPs is electrostatic [19], [20], we deduced that more than one class of aptamer could be simultaneously adsorbed onto the surface of AuNPs. Upon the addition of the corresponding target(s), the aggregation of the AuNPs could be induced, and the colloidal gold solution color would change from red to blue at a high salt concentration. Here we report an AuNP-based homogeneous colorimetric aptasensor for multiplex detection using sulfadimethoxine (SDM), kanamycin (KAN) and adenosine (ADE) as example targets. AuNP-based colorimetric aptasensors using SDM and KAN have been previously reported [17], [18]. These results demonstrate that an AuNP-based multiplex aptasensor can detect multiple combinations of the three targets with high sensitivity. The multiplex colorimetric aptasensor provides a powerful tool to examine a large number of samples for screening a small amount of potentially positive samples, which can further be validated by sophisticated instruments such as HPLC or LC-MS/MS.

## Materials and Methods

### Reagents and chemicals

The aptamer sequences used in this study were as follows:

SDM 5′-GAGGGCAACGAGTGTTTATAGA-3′ [21];

KAN: 5′-TGGGGGTTGAGGCTAAGCCGA-3′ [18];

ADE: 5′-ACCTGGGGGAGTATTGCGGAGGAAGGT-3′ [22].

The ssDNA oligonucleotides were synthesized by the Shanghai Sangon Biotechnology Co. Ltd. (Shanghai, China). The lyophilized powder was resuspended in 20 mM Tris-HCl containing 20 mM NaCl and 5 mM KCl and stored at 4°C until use. The oligonucleotide concentration was determined by measuring the UV absorbance at 260 nm. Chloroauric acid (HAuCl_4_) and sodium citrate (C_6_H_5_Na_3_O_7_) were obtained from Sigma-Aldrich (St. Louis, MO, USA). Sulfadimethoxine (SDM) and kanamycin (KAN) were purchased from Dr. Ehrenstorfer GmbH (Augsburg, Germany). Adenosine (ADE) was purchased from Amresco (Solon, OH). All remaining common chemicals, including NaCl, KCl and Tris-HCl, were purchased from the Beijing Chemical Reagent Company (Beijing, China). All chemicals were of at least analytical grade. A 96-well polystyrene microplate (12 strips of 8 wells) was purchased from Corning (Corning, NY). The water used in all experiments was purified using a Milli-Q system (Millipore, Bedford, MA, USA).

### Instrumentation

The ultraviolet-visible (UV-vis) absorption spectra of ssDNA and spectral measurements of the colloidal gold solution were performed using a NanoDrop 2000c Scan UV-vis spectrophotometer (Thermo Fisher Scientific Inc., Wilmington, USA) with a 10 mm path length fused-silica cuvette at room temperature.

### Synthesis of the citrate-protected AuNPs

AuNPs were synthesized using the classical citrate reduction method [23]. Briefly, colloidal AuNPs with an average diameter of 13 nm were prepared by rapidly injecting a sodium citrate solution (2 mL, 194 mM) into a boiling aqueous solution of HAuCl_4_ (100 mL, 1 mM) with vigorous stirring. After boiling for 20 min, the reaction flask was removed from the heat to allow the reaction solution to cool at room temperature. The concentration of the AuNPs was approximately 14 nM, which was determined according to Beer's law by using the extinction coefficient of 2.01×10^8^ M^−1^ cm^−1^ for 13 nm AuNPs in diameter at 520 nm [24].

### Procedure for multiplex detection

In a typical experiment, aptamers and targets were both dissolved in 20 mM Tris-HCl containing 20 mM NaCl and 5 mM KCl. Mixed aptamers (30 µL) were added into 70 µL target solution and incubated for 10 min. Then, 50 µL of AuNPs was transferred to the solution for a final volume of 150 µL and mixed thoroughly. After the solution was equilibrated for 10 min, the resulting solution was transferred to a 10 mm quartz cuvette. The UV–vis absorption spectrum was measured over the wavelength range from 450 nm to 750 nm with respect to water, and final photographs of the reaction cuvettes were taken with a Sony TX20 digital camera. All assays were performed at room temperature.

## Results and Discussion

**Figure 1:**
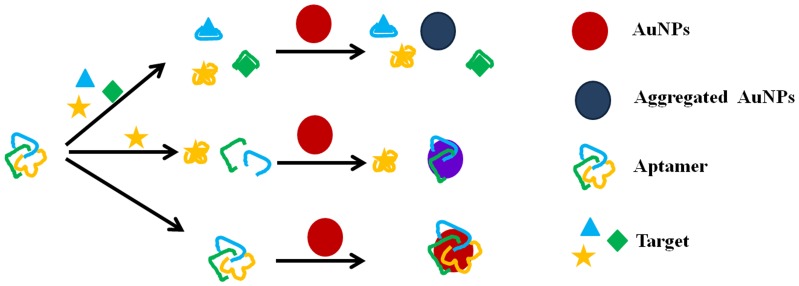
A schematic depicting the proposed multiplex detection using a colorimetric aptasensor.

**Figure 2:**
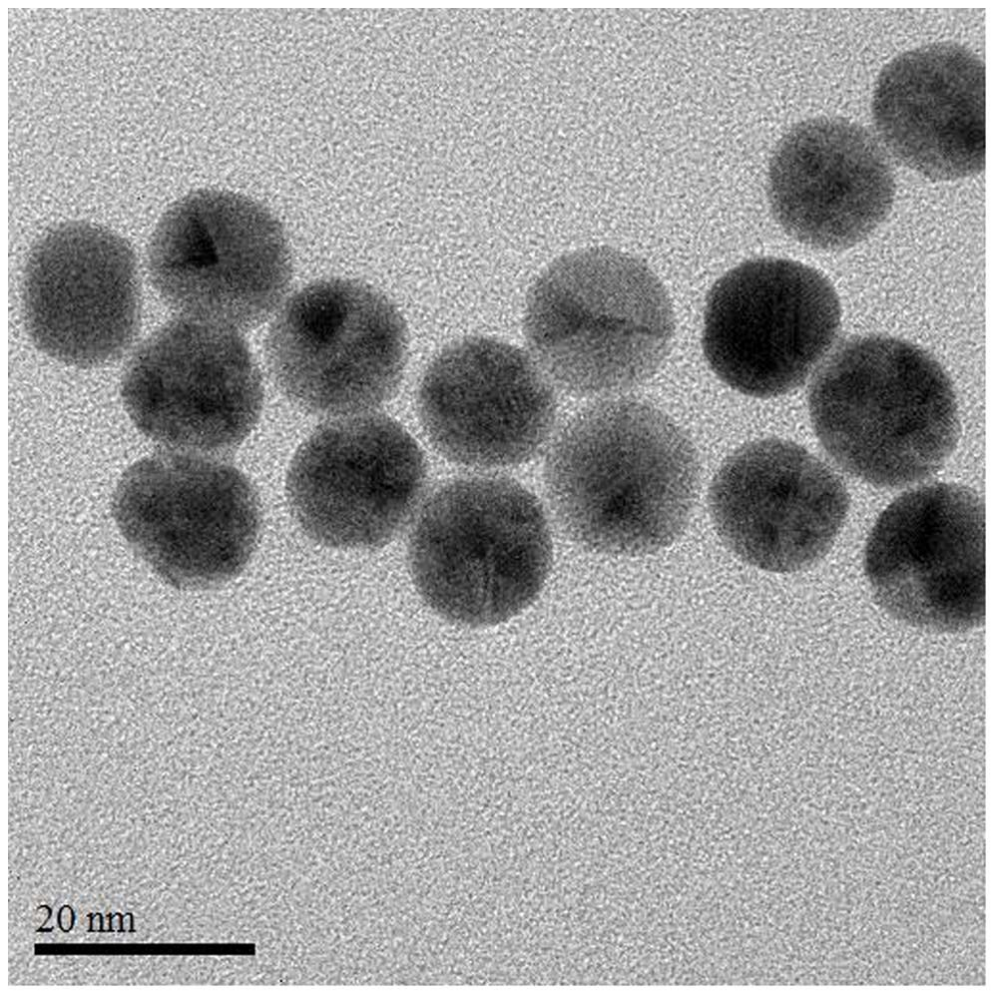
TEM image of AuNPs synthesized by sodium citrate.

A schematic of the multiplex detection principle is shown in Fig. 1. SDM, KAN and ADE aptamers were mixed at a particular ratio where they could adsorb onto the surface of unmodified 13 nm AuNPs (Fig. 2) in the absence of targets by the electrostatic interaction between bases of ssDNA and AuNPs. The electrostatic repulsion prevented the strong van der Waals attraction and enhanced the stability of AuNPs against buffer-induced aggregation, and thus the solution remained red in color. The ssDNAs cannot hybridize to each other to form double-stranded DNA, which are rarely adsorbed on AuNPs primarily due to the higher structural rigidity compared to ssDNAs. However, in the presence of targets, the conformation of an aptamer changes from a random coiled structure to a rigid folded structure after binding to targets. This transition induced the dissociation of aptamers from AuNPs and resulted in the subsequent aggregation of AuNPs, leading to the color change from red to purple/blue.

In this system, there are two key parameters that directly influence the performance of multiplex detection: the concentration of each aptamer and the buffer for the aptamer reaction with its target. An insufficient concentration of aptamer cannot stabilize AuNPs while an excess of aptamer will reduce the detection sensitivity because the targets compete with AuNPs for binding to a certain concentration of aptamer [17]. For simplicity, each aptamer concentration was selected as the minimum concentration to stabilize AuNPs and was determined as 750 nM for the KAN aptamer, 250 nM for the SDM aptamer and 500 nM for the ADE aptamer. All three aptamers were mixed at a 1∶1∶1 volume ratio, and the resulting mixed aptamers stabilized AuNPs efficiently. In order for all three aptamers to react with each target under similar conditions, a universal buffer (20 mM Tris-HCl containing 20 mM NaCl and 5 mM KCl) was used to dissolve the aptamers and targets. The proposed buffer alone is enough to induce AuNPs aggregation when the adsorbed aptamers are detached by binding to targets, making the aptasensor simpler and more convenient to use.

**Figure 3:**
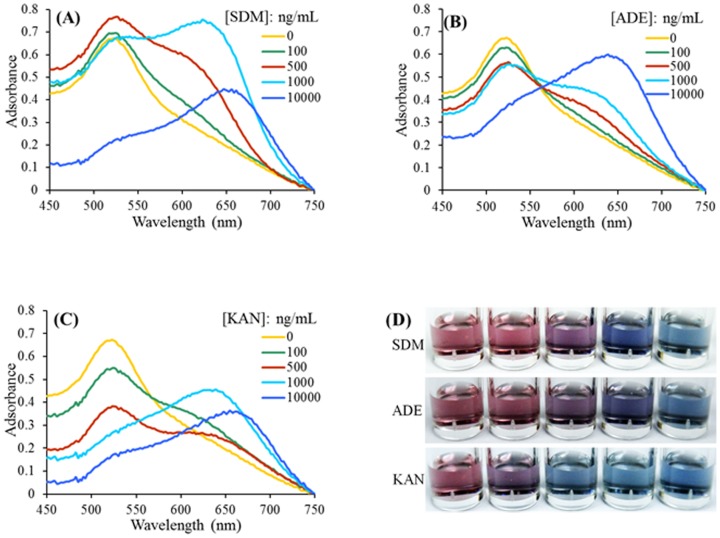
Absorption spectra of a single-detection aptasensor in the presence of various concentrations of corresponding targets: SDM (A), ADE (B), KAN(C). Corresponding photographic images (D).

Before the development of a multiplex system, we first developed three single aptasensors for SDM, KAN and ADE. In these single aptasensors, only one class of aptamer was used to stabilize the AuNPs, and the concentration of the aptamer was optimized as described above. The detection procedure was identical to the multiplex detection. As shown in Fig. 3, each individual aptasensor could successfully detect the corresponding target in a dose-dependent manner and reached a sensitivity of 500 ng/mL (ADE), 500 ng/mL (SDM) and 100 ng/mL (KAN) by visual inspection.

**Figure 4:**
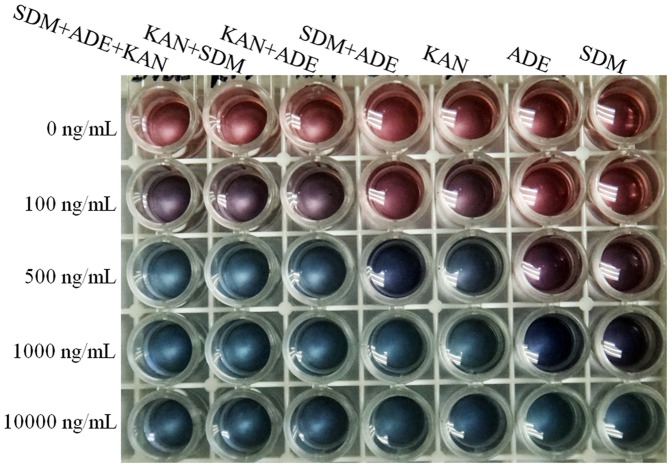
Photographic image of the multiplex aptasensor using different targets at different concentrations.

A colorimetric multiplex aptasensor was developed by mixing an equal volume of 750 nM KAN aptamer, 250 nM SDM aptamer and 500 nM ADE aptamer to stabilize the AuNP solution. To examine the multiplex aptasensor performance, we performed a series of experiments to detect a single target, two targets, or three targets. In these experiments, the target concentrations were set as 0, 100, 500, 1000 and 10000 ng/mL. For single-target detection using this multiplex aptasensor, as shown in Fig. 4, the system can easily detect each of the three targets with a sensitivity of 500 ng/mL (ADE), 500 ng/mL (SDM) and 100 ng/mL (KAN) by visual inspection. Therefore, the sensitivity is not reduced compared with the respective single-target aptasensor; three classes of aptamers were adsorbed onto the surface of AuNPs at 1/3 the concentration of the original single-target aptasensor.

**Figure 5:**
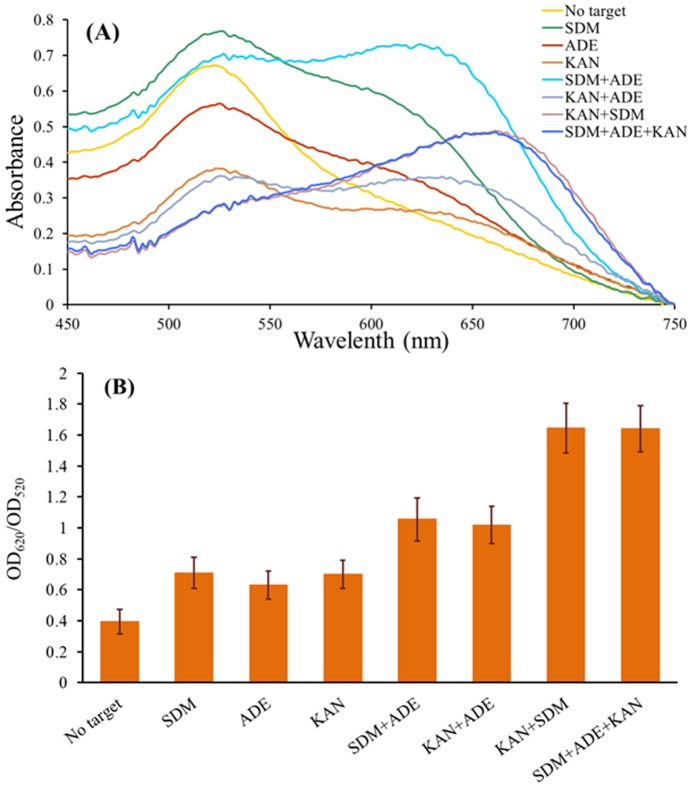
Absorption spectra of AuNPs solutions in the presence of different targets, each with a concentration of 500 ng/mL (A) and the corresponding ratio of 620 nm to 520 nm (B).

Using ADE and SDM as examples, the solution of AuNPs markedly changed color from red to purple at 500 ng/mL for both ADE and SDM. These results suggest that both 500 ng/mL ADE and 500 ng/mL SDM may induce AuNP aggregation by binding to the aptamers and desorbing the ssDNAs from the surface of the AuNPs, respectively. Accordingly, we predicted that there may be more ssDNA dissociation and AuNP aggregation if 500 ng/mL ADE and 500 ng/mL SDM were added simultaneously than if only one target were added to the aptamer mixture. By mixing 1000 ng/mL SDM with an equal volume of 1000 ng/mL ADE to make a two-target-containing sample, we tested the multiplex aptasensor for detection of both targets. As expected, the color of the AuNP solution changed to light blue, which combined both the SDM and ADE effects on the AuNPs at 500 ng/mL concentrations. The color changes can also be observed from the absorption spectra changes of AuNP solutions in the presence of different targets. As shown in Fig. 5, a higher ratio of 620 nm to 520 nm absorbance was obtained for ADE+SDM detection simultaneously than for detection of only one target at the same concentration. Accordingly, we hypothesized that the addition of the third target (KAN) to the system would produce a stronger color change and a higher ratio of 620 nm to 520 nm absorbance. Indeed, the color of the AuNP solution changed to strong blue when KAN, ADE and SDM were simultaneously added at 500 ng/mL for each target (Fig. 4). The ratio of the 620 nm to 520 nm absorbance for the KAN+ADE+SDM detection also increased to 1.64 from 1.05 for the SDM+ADE detection (Fig. 5). The same results were also obtained with the multiplex aptasensor for ADE+KAN and ADE+KAN+SDM or for SDM+KAN and SDM+KAN+ADE simultaneous detections. Notably, because KAN has a higher sensitivity and induced the color of the AuNP solution to light blue at 500 ng/mL, for KAN+SDM, the ratio may rise to the maximum that is the same as that for SDM+KAN+ADE (Fig. 5).

The multiplex aptasensor has its shortcomings, such that it cannot determine which targets are detected when a sample gives a positive result. However, in many cases, such as veterinary drug residue detection in animal production, a great deal of samples need to be screened for more than one antibiotic. A sensitive, fast and high-throughput screening method is required for which only a small number of samples with potentially positive results need to be confirmed using sophisticated instruments such as HPLC or LC-MS/MS. Therefore, if the same kind of aptamers, such various antibiotics or mycotoxin aptamers were used, the multiplex aptasensor may find application in food safety analysis for antibiotics residue screening or mycotoxins multiplex detection.

## Conclusions

In summary, we have successfully developed an AuNP-based colorimetric multiplex aptasensor by adsorbing more than one class of aptamer onto the surface of AuNPs. The use of a universal reaction buffer allowed for the omission of salt, which simplified the procedure. The multiplex aptasensor can detect more than one target simultaneously with the same sensitivity as a corresponding single-target aptasensor using SDM, ADE and KAN as example targets. As more aptamers are identified using SELEX technology for different targets, the multiplex aptasensor could be extended to other aptamers for various molecular detection events. Due to its simple design, easy operation, fast response, cost effectiveness and lack of need for sophisticated instrumentation, the proposed strategy may be applied to clinical diagnostics, food safety testing and environmental pollutant analysis.
